# Rising to the challenge: designing, implementing and reporting exercise oncology trials in understudied populations

**DOI:** 10.1038/s41416-020-0868-9

**Published:** 2020-05-21

**Authors:** Ian M. Lahart, Sarah K. Weller, Amy A. Kirkham

**Affiliations:** 1 Faculty of Education, Health and Wellbeing, Institute of Human Sciences, University of Wolverhampton, Wolverhampton, UK; 2 Graduate Program in Rehabilitation Sciences, University of British Columbia, Vancouver, BC Canada; 3 Department of Biomedical Engineering, University of Alberta, Edmonton, AB Canada

**Keywords:** Myeloma, Peer review

## Abstract

Exercise can improve cancer-related fatigue, quality of life and physical fitness, but is understudied in less common cancers such as multiple myeloma. Studying less common cancers and the adoption of novel study designs and open-science practices would improve the generalisability, transparency, rigour, credibility and reproducibility of exercise oncology research.

## Main

Exercise is an effective therapy for cancer-related fatigue^1^ and improving quality of life and physical fitness in patients with cancer.^2^ Most of this evidence, however, is in breast and prostate cancer populations.^2^ Multiple myeloma (MM), a haematological cancer associated with fatigue, muscle atrophy, reduced physical function and poor quality of life, is a population that could benefit from exercise rehabilitation.^3^ However, there is limited available evidence for the safety, feasibility and efficacy of exercise in MM.^3^ A primary barrier in implementing physical rehabilitation is the challenge imposed by the osteolytic bone lesions present in the majority of MM patients—with over half of patients experiencing pathological fracture or spinal cord compression.^4^

A recently published exercise guideline recommended selection of exercises that reduce load in areas with lesions in patients with metastatic bone disease.^2^ In MM, this approach is difficult to implement due to the extensive presence of osteolytic lesions throughout the body. The International Bone Metastases Exercise Working Group was recently established to develop guidelines that will provide further exercise guidance in metastatic bone disease, including recommendations for MM. An exercise guidance document is currently under development, with publication expected in 2020.

Given the increased risks and challenges of implementation of exercise in this population, Koutoukidis et al.^5^ in this issue of the *British Journal of Cancer* are to be commended on their study focussed on MM in this issue. The authors investigated the effects of a 6-month combined hospital-based supervised and home-based unsupervised aerobic and resistance exercise-training programme for MM survivors who had completed their initial treatment.^5^ Importantly, no exercise-related fractures or adverse events were reported among the 51 patients who participated in the exercise arm.

**Fig. 1:**
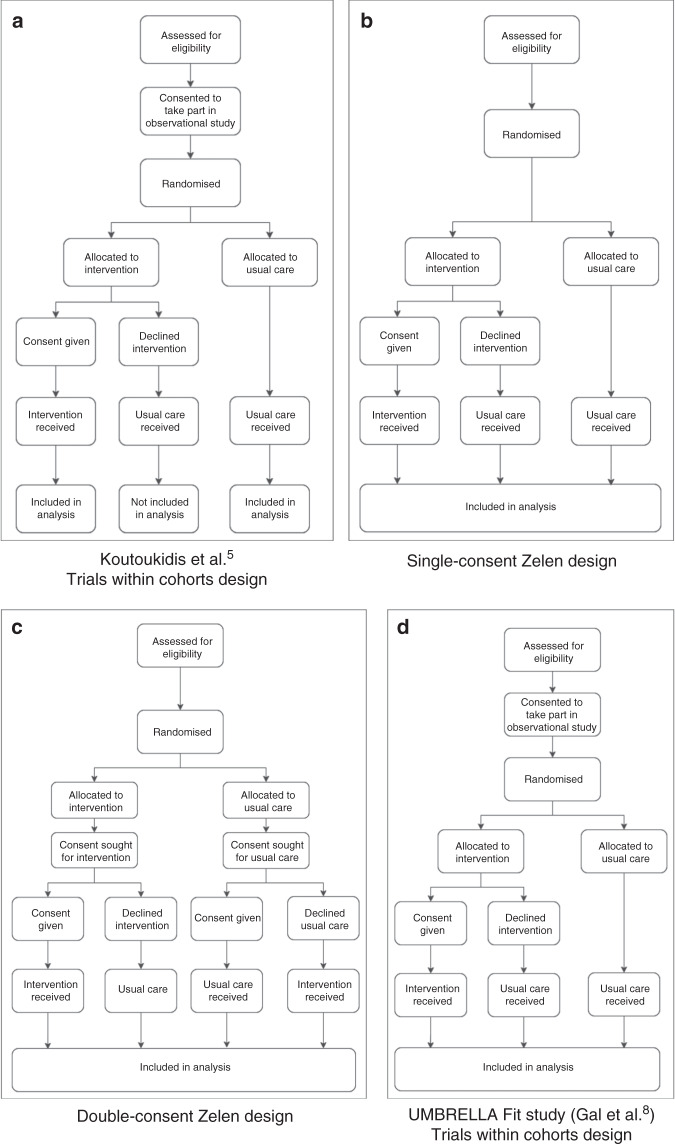
Diagrams of pragmatic study designs. **a** Trials within cohort design used in Koutoukidis et al.;^5^ **b** single-consent Zelen design; **c** double-consent Zelen design; **d** trials within cohort design used in Gal et al.^8^

In lieu of a traditional RCT design, the authors implemented an adapted Zelen design (more commonly known as ‘Trials within Cohorts’ [TwiC], Fig. 1a). Pragmatic trial designs, such as Zelen and TwiC approaches, are proposed to overcome control group contamination and study generalisability issues. In Zelen designs, consent is sought after randomisation either from patients who have been allocated to the intervention (single consent, Fig. 1b) or from patients in both intervention and usual care groups (double consent, Fig. 1c). While the aim is to reduce disappointment bias when patients are not allocated to their preferred treatment, remove subjective recruitment bias and minimise control group contamination,^6^ the ethics of Zelen designs have been questioned due to randomisation without consent and withholding treatment option information.^7^

In comparison, TwiC designs seek consent first from patients invited to participate in an observational trial, and secondly from patients within the cohort population if they are randomly allocated to an intervention (Fig. 1a, d). TwiC trials also aim to increase external validity by retaining characteristics of normal clinical practice.^7^ However, as observed by Koutoukidis et al.^5^ and a previous trial,^8^ TwiC studies in exercise oncology have reported high refusal rates of the intervention (43 and 48%, respectively),^5,8^ potentially resulting in underpowered analyses and dilution of intervention effects. This could have contributed to the lack of effect of the exercise intervention on fatigue, physical or emotional functioning, anxiety, depression or physical activity levels in the trial of Koutoukidis et al.^5^ This suggests that more education on the potential benefits of exercise may be required to enhance patient interest and the utility of these novel study designs.

While we appreciate the use of a novel study design and focus on an understudied, challenging population by Koutoukidis et al., the field of exercise oncology in general will benefit from further widespread adoption of a number of science reform practices. Recent Cochrane Collaboration systematic reviews of exercise oncology studies report widespread practices that reduce credibility and reproducibility of the results, including poor reporting standards, lack of prospective and/or detailed registration, low adherence to principles of exercise training, lack of blinding of outcome assessors and statisticians and underpowered statistical analyses.^9,10^

We believe that the adoption of a number of open-science practices would improve the rigour, transparency, credibility and reproducibility of exercise oncology research. For example, poor reporting practices can be relatively easily improved through diligent adherence to standard reporting guidelines (i.e., CONSORT-NPT, CERT) in the study design and reporting stages. Similarly, issues of exercise prescription design can be addressed through consideration of exercise-training principles^11^ and reporting guidelines (e.g., CERT). Adherence to and adoption of these guidelines requires a change in researcher behaviour and editorial policy, as well as editorial and peer-reviewer monitoring and scrutiny.

Prospective, detailed and transparent preregistration of trials can help solve a number of issues in exercise oncology. Preregistration helps to distinguish between confirmatory and exploratory research by making it clear which decisions (e.g., selection of primary outcomes and their analyses) were planned a priori (confirmatory) and which were made post hoc (exploratory).^12^ Preregistration can also be used to detect questionable research practices, such as selective outcome reporting, *p*-hacking and Hypothesizing After the Results are Known (HARKing).^12^ Lastly, preregistration can elucidate the prevalence and impact of publication bias and positive reporting bias by investigating how many and which planned trials are completed and published.

A promising and novel publishing format, Registered Reports, involves peer review and acceptance (for publication in principle) of preregistered proposals prior to data collection. A key benefit is that studies are judged on the relevance and importance of the research question, and the robustness and rigour of the trial design, and not on the study’s results. Registered Reports can also help to solve the problem of underpowered analyses by requiring confirmatory studies to be adequately powered. However, the challenge of acquiring the larger samples required to achieve sufficient statistical power and move exercise oncology beyond Phase 2–Phase 3 trials remains. One solution to this problem is to encourage multicentre collaborations. Indeed, to establish the benefits of exercise in patients with less common cancers such as MM, national or international collaborations are likely required.

In summary, to fully elucidate the effectiveness of exercise in the management of patients with cancer, we recommend the use of novel, pragmatic research designs, inclusion of less common cancer types and adoption of open-science practices.

## Data Availability

Not applicable.
